# Healthy lifestyle associated with dynamic progression of type 2 diabetes: A multi-state analysis of a prospective cohort

**DOI:** 10.7189/jogh.14.04195

**Published:** 2024-09-27

**Authors:** Yuanyuan Ma, Yufeng Chen, Aichen Ge, Guangfeng Long, Min Yao, Yanli Shi, Xiaowei He

**Affiliations:** 1 Department of Toxicology, School of Public Health, Qingdao University, Qingdao, China; 2 Department of Laboratory Medicine, People's Hospital of Rizhao, Rizhao, Shandong, China; 3 Department of Science and Technology, Children's Hospital of Nanjing Medical University, Nanjing, China; 4 Department of Clinical Laboratory, Children's Hospital of Nanjing Medical University, Nanjing, China; 5 Department of Stomatology, Children's Hospital of Nanjing Medical University, Nanjing, China; 6 Guangxi Medical University, Nanning, China

## Abstract

**Background:**

Although the association of a healthy lifestyle with type 2 diabetes (T2D) has been extensively studied, its impact on the dynamic trajectory, including progression, onset and prognosis, of T2D has not been investigated.

**Methods:**

Using data from the UK Biobank, 461 168 participants without diabetes or diabetes-related events were included. We incorporated four lifestyle factors to construct the healthy lifestyle score (HLS). We employed a multi-state model to examine the relationship between a healthy lifestyle and transition in T2D progression, including transitions from baseline to diabetes, complications, and further to death. The cumulative probability of above transitions based on the health lifestyle score was calculated.

**Results:**

The results indicated that adhering to 3–4 healthy lifestyles had an inverse association with the risk of transition from baseline to diabetes (hazard ratio (HR) = 0.966; 95% confidence interval (CI) = 0.935–0.998, *P* = 0.038), diabetes to complications (HR = 0.869; 95% CI = 0.818–0.923, *P* = 5.2 × 10^−6^), baseline to death (HR = 0.528; 95% CI = 0.502–0.553, *P* < 2 × 10^−16^, and diabetes to death (HR = 0.765; 95% CI = 0.591–0.990, *P* = 0.041) compared with maintaining 0–1 healthy lifestyles. In addition, the transition probability of the above transitions can be lower with maintaining 3–4 healthy lifestyles.

**Conclusions:**

Healthy lifestyles are negatively associated with the risk of multiple outcomes during the dynamic progression of T2D. Adherence to 3–4 healthy lifestyle behaviours before diabetes onset can lower the risk of developing T2D, further reducing the risk of diabetes complications and death in patients with T2D.

Type 2 diabetes (T2D), the most prevalent form of diabetes, accounted for 96% of all diabetes cases in 2021 [1]. An estimated 463 million individuals worldwide suffered from T2D in 2019, which resulted in 150 million deaths [2]. The incidence of T2D will continue to increase, with more than 590 million patients expected to be diagnosed with the T2D by 2035 [3]. With improvements in the management of diabetes, the patients are living longer and becoming susceptible to a different set of complications, including macrovascular conditions (coronary heart disease, stroke, and peripheral arterial disease) and microvascular conditions (diabetic kidney disease, retinopathy, and peripheral neuropathy). The incidence of cardiovascular and microvascular complications is 50 and 27%, respectively [4], thereby further increasing the risk of adverse outcomes, including mortality [5,6]. The multi-state outcomes of diabetes encompass the development of various conditions, including diabetes, diabetes-related complications, and mortality, throughout the progression of the disease. This dynamic progression entails transitions between various stages of diabetes [7,8]. However, there is a lack of understanding about the correlation between risk or intervention factors and the multi-state outcomes in the dynamic progression of T2D.

The American Diabetes Association (ADA) guidelines suggest that in addition to pharmacological interventions, the management of a healthy lifestyle is an essential approach for preventing and treating T2D [9]. Prior research has demonstrated that adopting a healthy lifestyle, which includes smoking cessation, moderate alcohol consumption, healthy dietary choices, moderate or intense physical activity, and maintaining a healthy weight, can delay the median onset of T2D by 3.96 years and extend the average lifespan by 1.44 years [10]. Furthermore, lifestyle modification (LSM) appears to be more effective than drug intervention in preventing T2D, as LSM reduces the risk ratio of T2D by 39%, compared to 36% for drug intervention [11]. Individuals with the healthiest lifestyles have a 75% lower risk of developing T2D than those with the least healthy lifestyles [12]. Among the lifestyle-related risk factors for T2D, physical inactivity is responsible for 7% of the disease burden [13], while obesity can increase the risk of T2D by a factor of 5.8 [14]. These findings collectively suggest that lifestyle plays a critical role in the risk of developing T2D and that maintaining a healthy lifestyle can lower this risk. However, the impact of a healthy lifestyle on the multi-state outcomes in the dynamic progression of T2D remains unknown, and the available research on this topic is limited.

In this study, we utilised data from the UK Biobank cohort and employed multi-state models to examine the relationship between a healthy lifestyle and various stages of T2D progression, including transition from baseline to diabetes, diabetes to complications, diabetes to death, baseline to death, and complications to death. A healthy lifestyle score, which incorporated smoking status, alcohol consumption, diet, and physical activity level, was used to assess the impact of a healthy lifestyle on diabetes progression. Moreover, we also calculated the cumulative probability of transition in different stages of T2D based on the health-lifestyle score. The findings of this study will provide evidence to reinforce the importance of healthy lifestyle management for T2D and enhance diabetes prevention efforts.

## METHODS

### Study design and population

The data for this study were derived from the UK Biobank, a large cohort study with previously described details [15]. The study enrolled half a million individuals between the ages of 40 and 69 at 22 assessment centres across the UK from 2006 to 2010. Demographic characteristics, socioeconomic status, lifestyle factors, and health-related outcomes were collected using questionnaires during the follow-up period. All participants provided informed consent, and the study was approved by the UK Biobank Ethics Committee.

A total of 461 168 subjects were included in the study. We excluded those with T2D (n = 1492) or cardiovascular disease (n = 9705) at baseline, and participants with diabetes-related events (diabetic eye disease, diabetic nephropathy, and diabetic neuropathy) before the diagnosis of T2D (n = 21 407). Individuals with missing data on healthy lifestyles were also excluded (n = 116). The population enrolment during the study is depicted in Figure S1 in the **Online Supplementary Document**.

### Healthy lifestyle score (HLS)

We constructed HLS using four lifestyle factors (smoking status, alcohol consumption status, physical activity level, and diet) as recommended by the ADA guidelines. Each factor was divided into healthy and unhealthy levels, and lifestyle-related data were obtained from self-reports at the baseline survey. The criteria for a healthy lifestyle were as follows: never smoking for smoking status; moderate drinking (1–14 g/d for women and 1–28 g/d for men) for alcohol consumption status; meeting at least five of the recommendations for dietary status (high intake of fruits, vegetables, whole grains, fish, shellfish, dairy products, and vegetable oils; reducing total intake of refined grains, processed meats, unprocessed meats, and sugar-sweetened beverages); and engaging in moderate-intensity physical activity ≥150 minutes/week or vigorous physical activity ≥75 minutes/week for physical activity level. The specific scoring criteria for each factor are presented in Table S1 in the **Online Supplementary Document**. Participants were assigned 1 point for each criterion they met for a healthy lifestyle and 0 for any other criterion. The final healthy lifestyle score ranged from 0 to 4, with higher scores indicating a healthier lifestyle. Self-reporting was used to collect lifestyle-related data during the survey.

### Ascertainment of outcomes

In this study, the key outcomes were T2D, T2D-related complications, and death. The outcome data were obtained from admission, primary care, and death records provided by the National Health Service. T2D and T2D-related complications were coded according to the International Classification of Diseases, 10th Revision (ICD-10). The codes used for different diseases in this study were as follows: diabetes mellitus (E11); diabetic ophthalmopathy (E11.3, H36.0, H28.0); diabetic neuropathy (E11.4, G99.0); diabetic nephropathy (E11.2, E18.0, E18.3-5, N08.3); peripheral vascular disease (E11.5, I73.8, I73.9); cardiovascular disease (I21, I22, I23, I63, I64, I20.0, or OPSC4 codes K40, K41, K42, K43, K44, K45, K46, K483, K49, K501, K75, and K76); diabetes with coma (E11.0); ketoacidosis (E11.1); and other specific (E11.6) or nonspecific (E11.8) complications.

### Covariates

To account for potential confounders that could impact the association between healthy lifestyle and diabetes outcomes, we adjusted for several variables, including age (years), sex (male/female), race (white or other), body mass index (BMI) (<25kg/m^2^, 25 to 29.9kg/m^2^, ≥30kg/m^2^), baseline cancer prevalence (yes/no), low density lipoprotein (LDL) levels (continuous), and high-density lipoprotein (HDL) levels (continuous). The baseline questionnaires or self-reports, as well as admission data, were used to obtain these covariate data. BMI was calculated as the ratio of weight to height squared, with obesity defined as ≥30kg/m^2^. Non-fasting venous blood samples were collected from the participants and stored at -80°C after processing. The concentrations of LDL and HDL in blood (millimoles per litre (mmol/L)) were determined by blood biochemical analysis, and the last diet and water intake were recorded.

### Statistical analysis

**Figure 1:**
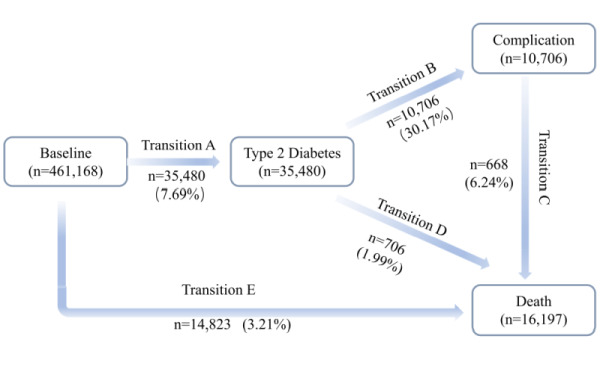
Numbers of participants in five transitions from baseline to T2D, T2D complications, and all-cause death. The T2D complications involved in this study included diabetic eye diseases, diabetic kidney diseases, diabetic neuropathy diseases, cardiovascular diseases, peripheral vascular diseases, and metabolic events. T2D – type 2 diabetes.

In this study, baseline characteristics of the study population were stratified by HLS. The age distribution was reported as the mean ± standard deviation (SD), while the distribution of healthy lifestyle factors and related covariates were presented as percentages (%). For the primary analysis, a multi-state regression model was used to estimate the HR and 95% CI for the association between HLS and various stages of T2D progression. The multi-state regression model is an extension of traditional Cox and Markov competing risk models, and is used to investigate the association between exposure factors and various stages of disease progression. Earlier studies have used multi-state models to explore the connection between environmental pollutants and the dynamic progression of diabetes and cardiovascular diseases [8,16]. Based on these studies, we considered five transition stages in the dynamic progression of T2D (**Figure 1**):

a) baseline to T2D;

b) T2D to any diabetes-related complication;

c) complication to death;

d) T2D to death;

e) baseline to death (no T2D).

For the population with more than one state occurring in the same period, we defined the start time of the former state as the start time of the latter state minus the median interval of each phase in the study (median interval: 3.85 years in phase b; 1.15 years in phase c; and 1.29 years in phase d).

Missing data for continuous variables, such as LDL concentration and HDL concentration, were imputed using multiple imputation with chained equations (30 409 and 66 135 individuals with missing data on LDL and HDL respectively). Two models were constructed to assess the association between a healthy lifestyle and the five transition stages in the dynamic progression of T2D while adjusting for covariates in the study. In model 1, we adjusted for age, sex, and race. Model 2, on the other hand, adjusted for additional covariates such as BMI, low-density lipoprotein, high-density lipoprotein, and baseline cancer prevalence, in addition to age, sex, and race. We considered model 2 as the main model in subsequent analyses to minimise the possibility of bias in our study. In addition, we performed a likelihood ratio test to assess the nonlinear association between HLS and cumulative transition probabilities for each stage of T2D progression. We further estimated the predicted cumulative transition probabilities for each stage of T2D progression among men and women with different HLS stratified by age (above and below the median age of 57 years), adjusting for other covariates such as race, BMI, baseline cancer prevalence, LDL, and HDL. To increase the sample size for the probabilistic prediction model, we adjusted the covariates based on the largest distribution within each group.

We performed sensitivity analyses to evaluate the robustness of our findings. The analyses included the following:

a) excluding CVD deaths (n = 2749);

b) excluding subjects with a follow-up time of less than two years (n = 2087);

c) excluding subjects with missing covariate data (n = 29 766);

d) computing the exposure-effect association between the HLS and the transition stages of dynamic progression of diabetes.

Statistical analyses were conducted using *R* version 4.1.3 (R Foundation for Statistical Computing, Vienna, Austria) and Stata MP version 16 (SAS Institute, Cary, NC). The multistate model was implemented using the ‘mstate’ package. All tests were two-tailed, and *P*-values <0.05 were considered statistically significant.

## RESULTS

### Baseline characteristics

During a median follow-up of 7.6 (IQR = 6.9–8.2) years for diabetes, a total of 35 480 participants developed T2D, of whom 10 706 developed T2D complications and 668 died from T2D complications. The specific characteristics of each transition state in the dynamic progression of T2D are illustrated in **Figure 1****.** **Table 1** presents the baseline characteristics of the study population stratified by HLS. The mean age of the study population was 56.2 years (SD = 8.1 years), with 44.1 male and 95% white race. Baseline characteristics of individuals with different HLS indicated a higher proportion of obese people (BMI≥30 kg/m^2^) in those with lower scores (0–1). Never smokers accounted for 55.5% of the study population, and this proportion increased with an increase in HLS. However, current drinkers accounted for 92.4% of the study population. The prevalence of hypertension and cancer was 19.1 and 7.6%, respectively. According to the Townsend Deprivation Index, we observed that individuals with higher healthy living scores (3–4 scores) had a higher socioeconomic status than those with lower scores (0–1 scores) in this study.

**Table 1:** Characteristics of 461 168 participants

Variables	Overall (n = 461 168)	Healthy lifestyle score			*P*-value
		**0–1 point**	**2 points**	**3–4points**	
Age in years, mean (SD)	56.2 (8.1)	56.2 (8.1)	56.1 (8.1)	56.4 (8.1)	<0.001
Sex, %					<0.001
*Female*	55.9	54.8	56.3	56.6	
*Male*	44.1	45.2	43.7	43.4	
Race, %					<0.001
*White*	94.6	94.4	94.3	95.1	
BMI, %					<0.001
*Normal (<25 kg/m^2^)*	34.5	29.4	34.1	40.7	
*Overweight (25–29.9 kg/m^2^)*	42.7	42.3	43.1	42.5	
*Obesity (≥30 kg/m^2^)*	22.4	27.7	22.3	16.5	
*Missing*	0.4	0.6	0.5	0.3	
Townsend deprivation index	−1.4	−0.9	−1.4	−1.9	<0.001
Smoking status, %					<0.001
*Never*	55.5	22.5	59.8	88.1	
*Past*	33.7	55.0	32.8	10.4	
*Current*	10.4	21.8	7.2	1.4	
*Missing*	0.4	0.7	0.2	0.1	
Alcohol drinking, %					
*Never*	4.2	4.5	4.8	3.1	<0.001
*Past*	3.3	5.1	3.2	1.4	
*Current*	92.4	90.2	91.9	95.5	
*Missing*	0.1	0.2	0.1	0.00	
Hypertension, %					<0.001
*Yes*	24.1	73.2	76.1	78.7	
*No*	75.9	26.8	23.9	21.3	
Cancer, %					<0.001
*Yes*	7.6	8.0	7.5	7.4	
*No*	92.1	91.5	92.3	92.4	
*Missing*	0.3	0.5	0.2	0.2	

BMI – body mass index, SD – standard deviation

### Association of HLS with different progressions of T2D

The associations between HLS and the risks of dynamic progression to T2D are presented in **Table 2**. In the basic model, the HLS was significantly associated with four transition stages in T2D progression, namely, T2D to complications, complications to death, and T2D to death. After adjusting for covariates in model 2, we observed that participants who adhered to 3–4 healthy lifestyles had a lower risk of transition from baseline to T2D, T2D to complications, baseline to death, and T2D to death compared to those who adhered to 0–1 healthy lifestyles. Specifically, the hazard ratio (HR) of transition from baseline to T2D was 0.966 (95% CI = 0.935–0.998, *P* = 0.038), and the HR of transition from T2D to complications was 0.869 (95% CI = 0.818–0.923, *P* = 5.2 × 10^−6^). Moreover, the HR of transition from T2D to death was 0.765 (95% CI = 0.591–0.990, *P* = 0.041). However, we did not observe a significant association between HLS and progression from diabetic complications to death (HR = 0.849, 95% CI = 0.636–1.134, *P* = 0.268).

**Table 2:** Associations between healthy lifestyle and risk of five progressions of type 2 diabetes using the multi-state model

Transition	Healthy life score		
	**0 − 1 points**	**2 points**	**3 − 4 points**
Basic model*			
*Baseline → diabetes*	1.0	1.004 (0.980,1.029)	1.007 (0.981,1.035)
*Baseline → death*	1.0	0.687 (0.662, 0.712)‡	0.540 (0.518, 0.564)‡
*Diabetes → complication*	1.0	0.849 (0.813, 0.887)‡	0.741 (0.706, 0.779)‡
*Diabetes → death*	1.0	0.670 (0.562, 0.797)‡	0.713 (0.587, 0.865)‡
*Complication → death*	1.0	0.794 (0.667, 0.945)‡	0.683 (0.542, 0.861)‡
Model 2†			
*Baseline → diabetes*	1.0	0.981 (0.951, 1.011)	0.966 (0.935, 0.998)‡
*Baseline → death*	1.0	0.681 (0.654, 0.709)‡	0.528 (0.504, 0.553)‡
*Diabetes → complication*	1.0	0.906 (0.858, 0.957)‡	0.869 (0.818, 0.923)‡
*Diabetes → death*	1.0	0.752 (0.595, 0.951)‡	0.765 (0.591, 0.990)‡
*Complication → death*	1.0	0.886 (0.706, 1.113)	0.849 (0.636, 1.134)

*Basic model was adjusted for age, sex, and race.

†Model 2 was based on a basic model and additionally adjusted for body mass index, cancer, high-density lipoprotein, and low-density lipoprotein.

‡*P* < 0.05, all values are statistically significant (*P* < 0.05).

### Cumulative transition probabilities for stages of T2D progression

**Figure 2:**
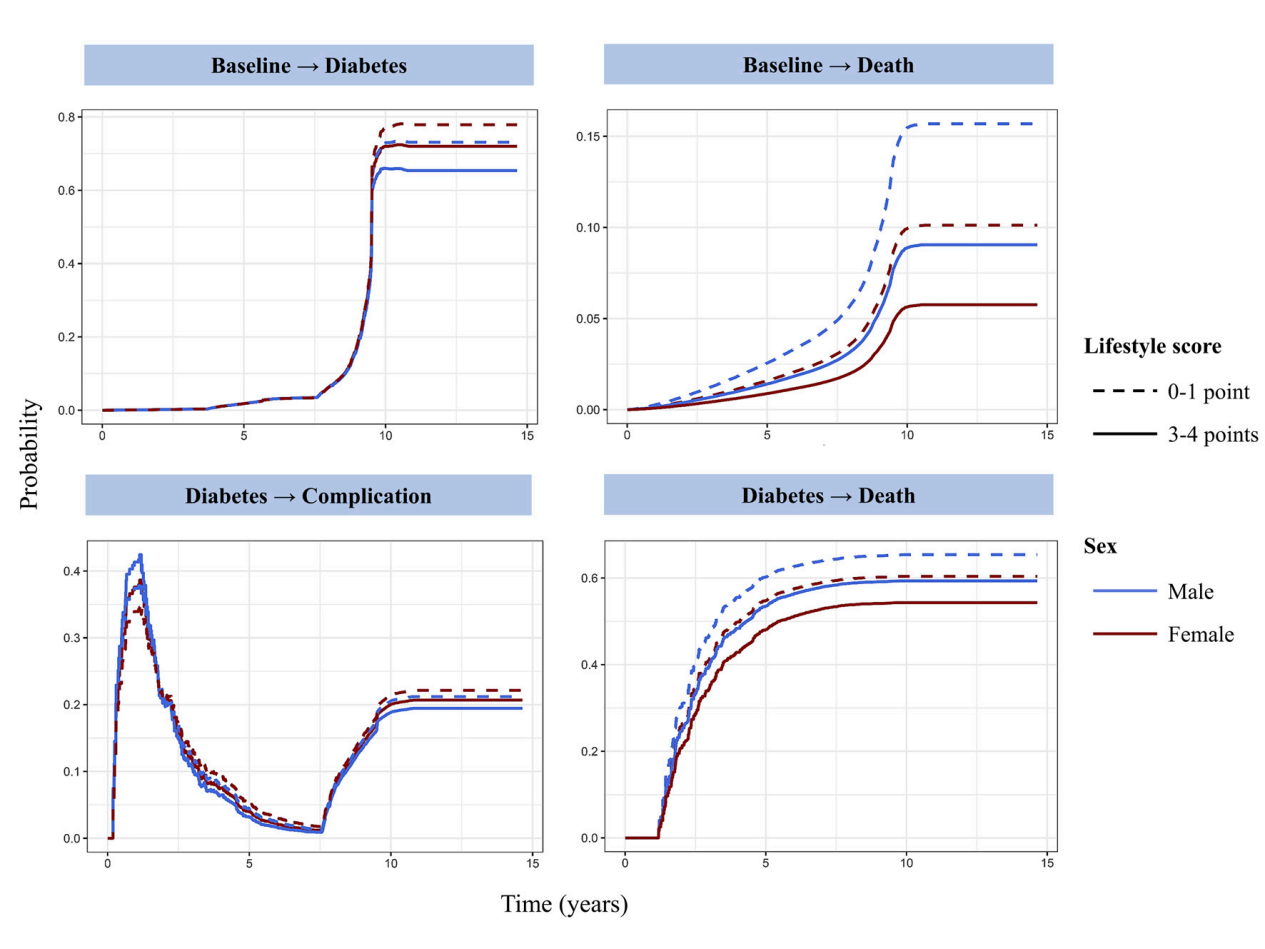
Cumulative transition probabilities of type 2 diabetes for participants with different healthy lifestyle. Computed for age >57 years old men and women in lifestyle score 3–4 points (continuous) and 0–1 point(dotted). The model was adjusted for age, sex, race, body mass index, cancer, high-density lipoprotein, and low-density lipoprotein.

**Figure 2** illustrates the cumulative transition probabilities of the four diabetes transfer states related to the HLS during the follow-up period for individuals aged 57 years or older, stratified by different healthy lifestyle scores. During the 14.9 years of follow-up, individuals with lower HLS had higher probabilities of transition to T2D, T2D complications, or death outcomes. For men and women with a low lifestyle score (0–1 point), the probability of transition from healthy to T2D was 73.12 and 77.86%, respectively. This probability decreased to 65.34 and 72.02% for those with a higher HLS (3–4 points). Compared to individuals who adhered to three or four healthy lifestyles, those who adhered to one or fewer healthy lifestyles had a cumulative transition probability of T2D to T2D complications that was approximately 1.5% higher during the 14.9 years of follow-up (21.20% in 0–1 point vs. 19.44% in 3–4 point for men and 22.13% in 0–1 point vs. 20.67% in 3–4 point for women). The probability of transition from baseline to death was higher in men than in women. Participants with higher HLS had lower transition probabilities from baseline to death (15.69% in 0–1 point vs. 9.05% in 3–4 point for men, and 10.12% in 0–1 point vs. 5.76% in 3–4 point for women). During the 14.9 years of follow-up, maintaining three or four healthy lifestyle factors was associated with about 6% cumulative transition probability reduction in T2D to death transition (65.39% in 0–1 point vs. 59.36% in 3–4 point for men, and 60.41% in 0–1 point vs. 54.17% in 3–4 point for women). However, there was no significant difference in the cumulative transition probability from diabetic complications to death. The cumulative transition probabilities for the four transition states in the dynamic progression of T2D associated with a healthy lifestyle were consistent for those younger and older than 57 years, indicating that maintaining three to four healthy lifestyles reduced the transition probability of the four transition states (baseline to T2D, T2D to complications, baseline to death, and T2D to death) compared to those who maintained a 0–1 healthy lifestyle (Figure S2 in the **Online Supplementary Document**). However, the HLS was not associated with the transition from diabetic complications to death, and there was no significant difference in the transition probability between the population with 3–4 healthy lifestyles and the population with 0–1 healthy lifestyles at this stage (Figure S3 in the **Online Supplementary Document**).

### Sensitivity analyses

Our results were confirmed to be robust through sensitivity analyses. After excluding individuals who had died from cardiovascular disease or had been followed for less than two years, the association between a healthy lifestyle and the risk of progression to the four categories of diabetes (baseline to diabetes, diabetes to complications, baseline to death, and diabetes to death) remained consistent with the findings in model 2 above. Furthermore, our results remained consistent after excluding individuals with missing covariates. These sensitivity analyses indicate that a healthy lifestyle may impact the other four stages of diabetes progression, except for diabetes complications to death. Table S2 in the **Online Supplementary Document** presents the results of these sensitivity analyses. In this study, we also examined the exposure-effect associations of a healthy lifestyle with each transition state in the dynamic progression of diabetes using models 1 and 2. The results revealed that the inverse association between a healthy lifestyle and T2D multi-state outcomes persisted even after adjusting for relevant covariates in model 2. With every point increasing of HLS, the HRs for different transitions in dynamic progression of T2D were displayed in Table S3 in the **Online Supplementary Document**.

## DISCUSSION

The study findings suggest that a healthy lifestyle is associated with a reduced risk of transition among the multi-state outcomes of T2D, T2D complications, and death during the dynamic progression of T2D. Specifically, maintaining 3–4 healthy lifestyles was found to lower the risk of transition during the progression of T2D. The cumulative transition probability of dynamic progression of diabetes decreased with the increasing of HLS, particularly the transition of baseline to T2D, T2D to complication, and T2D to death. These results underscore the importance of managing a healthy lifestyle during the dynamic progression of T2D and provide evidence for the prevention and treatment of T2D at various stages of progression.

Healthy lifestyle management is a crucial method for preventing and treating T2D [17]. Multiple prospective cohort studies have demonstrated that healthy lifestyle interventions significantly reduce the risk of T2D, with risk ratios ranging from 0.32 to 0.71 [18–20]. Consistent with previous studies, our findings indicate that a healthy lifestyle is associated with a reduced risk of transition to T2D. Specifically, the HR of participants with a healthy lifestyle score of 3–4 was 0.966 (95% CI = 0.935–0.998) compared to those with a score of 0–1. The considerable variation in the findings across studies may be due to differences in the study population or the factors involved in healthy lifestyle.

While some studies have focused on the onset of T2D, few have explored the factors influencing the dynamic progression of diabetes. A study categorised the progression of diabetes into stages, based on the underlying pathophysiological processes, including the euglycemic with hyperinsulinemia stage, the prediabetic stage. The results demonstrated that managing obesity can delay the progression of diabetes [21]. However, most current studies on the relationship between healthy lifestyle and diabetes focus on single disease states, such as diabetes or diabetes complications [22,23], without considering multi-state outcomes and transfer processes in the dynamic progression of diabetes. In our study, we found that adherence to 3–4 healthy lifestyles reduced the transition probability of baseline to diabetes, diabetes to diabetes complications, and diabetes to death compared to those who maintained 0–1 healthy lifestyles. These findings emphasise the importance of maintaining a healthy lifestyle for the prevention and management of T2D at different stages of progression.

Diabetes complications are the leading cause of are disability and mortality [24]. Advances in the treatment of T2D mean that people with diabetes are living longer than they used to, further increasing the incidence of diabetes complications and the treatment cost, as more than half of the cost of diabetes treatment is derived from the treatment of complications [25]. A cohort study from the UK Biobank indicated that adhering to a healthy lifestyle can reduce the incidence of cardiovascular disease in people with diabetes. The most significant effects were observed for noncentral obesity and non-smoking, with hazard ratios of 0.67 (95% CI = 0.55–0.81) and 0.70 (95% CI = 0.58–0.83), respectively [26]. Other studies have demonstrated that adherence to 4–5 healthy lifestyle factors, including noncentral obesity, no smoking, moderate alcohol consumption, active physical activity, and a healthy diet, can lower the hazard ratio for diabetic retinopathy to 0.65 (95% CI = 0.46–0.91), diabetic nephropathy to 0.43 (95% CI = 0.30–0.61), and diabetic neuropathy to 0.46 (95% CI = 0.29–0.74) [27]. Although the types of diabetes complications were not separately analysed in this study, our results demonstrate that adhering to 3–4 healthy lifestyle factors, compared to 0–1 healthy lifestyle factors, was associated with a reduced risk of complications in patients with diabetes (HR = 0.869; 95% CI = 0.818–0.923, *P* = 5.2 × 10^−6^). Therefore, our findings suggest that a healthy lifestyle can play an important role in reducing the risk of complications in patients with T2D.

Previous researches have established a strong correlation between lifestyle and all-cause mortality risk among diabetes patients, with the risk ratios ranging from 0.42 to 0.58 [28–30]. Consistent with previous studies, our results showed that a healthy lifestyle was associated with a reduced hazard ratio for the transition from diabetes to all-cause mortality (HR = 0.765; 95% CI = 0.591–0.990, *P* = 0.041). The protective effect of a healthy lifestyle on transition status was smaller than that from baseline to all-cause mortality (HR = 0.528; 95% CI = 0.502–0.553, *P* < 2 × 10^−16^). This finding suggests that interventions aimed at promoting a healthy lifestyle prior to the onset of diabetes may result in a greater reduction in the risk of death. However, there was no significance association of HLS with the transition from complication to death. This may be due to the fact that the types of complications included in this study are not the leading causes of death. Besides, the gender difference was observed in the association of HLS with dynamic progression of diabetes. With the same HLS, the cumulative transition probability of diabetes dynamic progression appears to be higher in women than in men.

This phenomenon is consistent with the relatively large number of female diabetes patients in the world [31]. The reason may be that women are more vulnerable to impaired postprandial glucose tolerance or vitamin D deficiency [31,32]. Further research is needed to clarify the specific mechanism.

Currently, the biological mechanisms underlying the association between a healthy lifestyle and diabetes or diabetic complications are not well understood. Islet dysfunction is a known cause of diabetes [33]. Studies have shown that 3–6 months of lifestyle intervention can significantly improve diabetes, potentially by improving insulin resistance or islet β cell function [34]. Exercise can augment glucose disposal and increasing insulin sensitivity, thus can be a tool to aid in glucose regulation [35,36]. Smoking cessation can reduce the changes in body composition, decreased insulin sensitivity and decreased islet function caused by nicotine and other exposures [37]. Chronic inflammation is a pivotal pathophysiological process in the development of diabetes and diabetic complications [38]. Unhealthy lifestyles, such as insufficient sleep, high-fat diets, and increased BMI, have been linked to the promotion of inflammatory factors or inflammatory immune mediators in the body [39–41]. Conversely, adopting a healthy lifestyle, such as engaging in regular physical exercise, has been shown to alleviate chronic inflammation and reduce the risk of developing diabetes [42,43]. These findings suggest that a healthy lifestyle may mitigate the progression of diabetes by reducing inflammatory factors or slowing the inflammatory process. Furthermore, adopting a healthy lifestyle may significantly reduce the risk of microvascular and cardiovascular complications in patients with T2D through metabolomic changes by improving blood glucose control, systemic inflammation, liver function, and lipid-related metabolites.

Our study has several strengths. First, we utilised a large sample size from the UK Biobank with complete demographic, anthropometric, and disease-related data to construct a multistate model of the dynamic progression of diabetes. Second, we are the first to investigate the association of a healthy lifestyle with the dynamic progression of diabetes, including the transition from a healthy state to diabetes, complications, and ultimately death. Third, we used a multi-state regression model instead of the traditional Cox regression model to analyse the risk association of a healthy lifestyle with the five metastatic stages in the dynamic progression of T2D. Fourth, we conducted multiple sensitivity analyses to assess the robustness of our results and adjusted for potentially influential covariates. Through our study, we identified the critical stages in the progression of diabetes and the impact of a healthy lifestyle on this process.

However, our study has several limitations that should be acknowledged. First, the lifestyle-related data were collected through self-reported measures at baseline, which may not fully reflect changes in participants' lifestyle habits after the diabetes diagnosis, leading to an underestimation of the observed association. Second, the outcome definition of T2D complications was based on the ICD-10, which may result in some of the complications not being specific to T2D. Thus, it may result in misclassification bias. Third, our study sample was derived from the UK Biobank, which primarily consists of individuals of white ethnicity with different diets and lifestyle habits than those of other regions, limiting the generalisability of the findings.

## CONCLUSIONS

Give main conclusions of your study. Our study found a negative correlation between a healthy lifestyle and the risk of multiple outcomes during the dynamic progression of T2D, indicating that adherence to 3–4 healthy lifestyle behaviours before T2D onset can lower the risk of developing T2D. After T2D diagnosis, maintaining 3–4 healthy lifestyle behaviours not only reduces the risk of T2D complications but also lowers the risk of death, thereby potentially extending lifespan compared to individuals who maintain 0–1 healthy lifestyle behaviour. These findings suggest the importance of healthy lifestyle management in preventing T2D and diabetic complications during the dynamic progression of T2D.

## Additional material


Online Supplementary Document
