# Supplementary material for: Healthy lifestyle associated with dynamic progression of type 2 diabetes: A multi-state analysis of a prospective cohort
Source: J Glob Health. 2024 Sep 27;14:04195. doi: 10.7189/jogh.14.04195 (PMC11427933; doi:10.7189/jogh.14.04195)
Supplement: Online Supplementary Document [file jogh-14-04195-s001.pdf]

Supplementary Materials

Table of content

Figure S1 Flowchart of study participant selection and analysis process.
Figure S2 Cumulative transition probabilities of diabetes for participants with different healthy lifestyle score.
Figure S3 Cumulative transition probabilities of diabetes complication to death for participants with different healthy lifestyle score.

Table S1 Definition and healthy level of each factor in healthy lifestyles.
Table S2 Sensitivity Analysis
Table S3 Exposure-response associations between healthy lifestyle and different transitions of diabetes.

Supplementary Figures

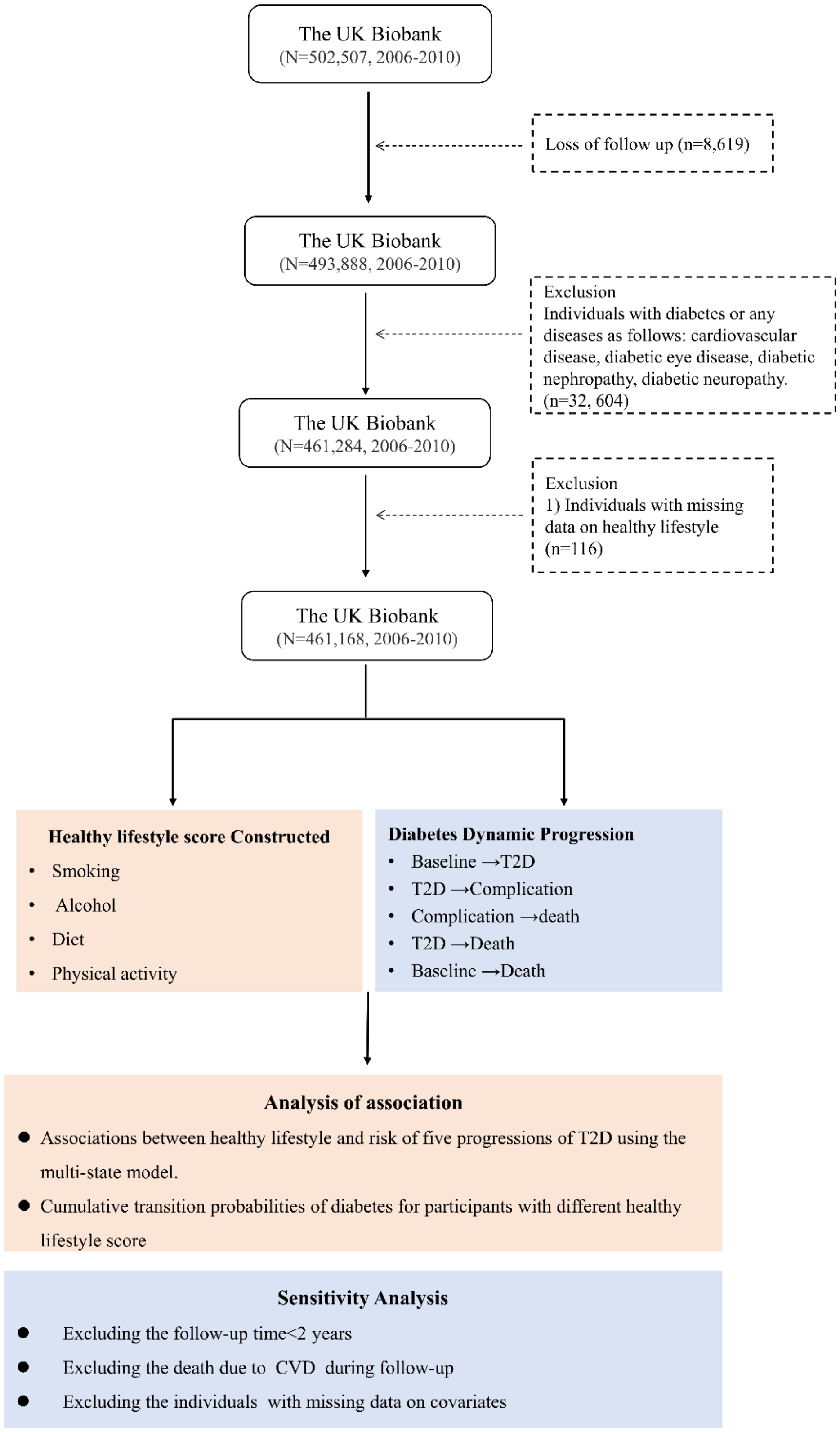

Figure S1 Flowchart of study participant selection and analysis process.

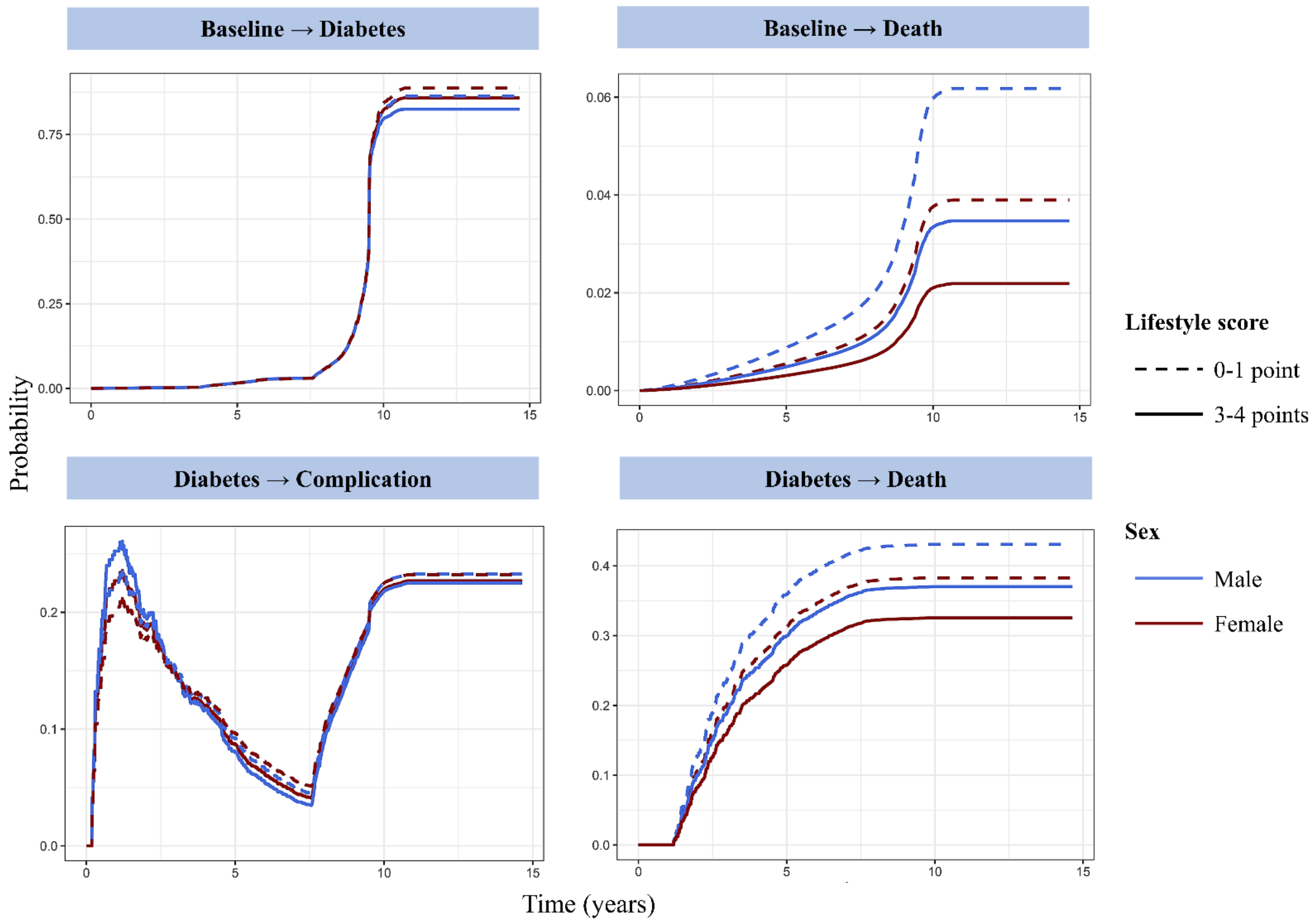

Figure S2 Cumulative transition probabilities of diabetes for participants with different healthy lifestyle score. Computed for age<57 years old men and women in lifestyle score 3-4 points (continuous) and 0-1 point(dotted). The model was adjusted for age, sex, race, BMI, cancer, HDL, and LDL.

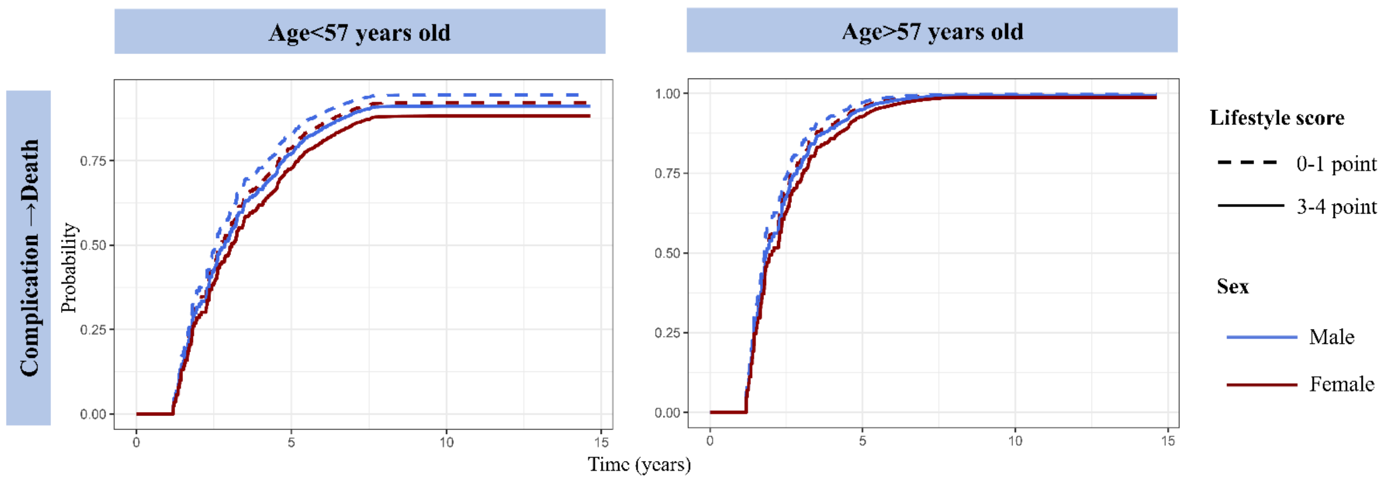

Figure S3 Cumulative transition probabilities of diabetes complication to death for participants with different healthy lifestyle score. Computed for age<57 years and age>57 years old men or women in lifestyle score 3-4 points (continuous) and 0-1 point(dotted). The model was adjusted for age, sex, race, BMI, cancer, HDL, and LDL.

Supplementary Tables

Table S1 Definition and healthy level of each factor in healthy lifestyles.

**Healthy lifestyle factor**	Healthy level **(Assigned 1 point)**	**Self-reported UK Biobank field code**
Smoking status	Smoking status was defined as current, previous, never smoker; Never smoking was considered as a healthy level	20116
alcohol consumption	Participants were asked about the frequency of drinking alcohol, i.e., (almost) daily, three or four times a week, once or twice a week, one to three times a month, special occasions only, never, and prefer not to answer. Those who reported to drink alcohol would be asked about how much red wine (glasses), white wine (glasses), beer or cider (pints), spirits or liqueurs (standard measures), fortified wine (glasses), and other alcoholic drinks (glasses) they consumed in an average month or week. We used the information to calculate the average units of alcohol each participant drank daily.<br>A healthy level was defined as daily consumption of one drink or fewer for women and two drinks or fewer for men, according to the dietary guidelines in the US and UK (one drink contains 14 g of ethanol in the US and 8 g in the UK)	20117
physical activity	≥ 150 minutes moderate activity per week OR ≥ 75 minutes vigorous activity per week OR equivalent combination OR moderate physical activity at least 5 days a week and vigorous activity once a week.	884, 894, 904, 914
Healthy diet	At least 5 of the following recommendations:<br>1. Fruits: ≥ 3 servings/day<br>2. Vegetables: ≥ 3 servings/day<br>3.Whole grains: ≥ 3 servings/day<br>4. (Shell)Fish: ≥2 times/week<br>5.Diary: ≥ 2 servings/day<br>6.Vegetables oils: ≥ 2 servings/day<br>7.Refined grains: ≤2 servings/day<br>8. Processed meats: ≤ 1 servings/week<br>9. Unprocessed meats: ≤ 2 servings//week<br>10. Sugar-sweetened beverages: Don't drink<br><br>(Amount per serving: fresh fruit- 1 piece; dried fruit- 5 pieces; vegetables- 3 heaped tablespoons; whole meal/wholegrain bread- 1 slice/day); bran/oat/muesli cereal 1 bowls/week; oily fish/non-oily fish	1309, 1319, 1289, 1299, 1438,1448,1458,1468, 1329,1339,1408,1418, 1428,2654,1349,3680, 1359,1369,1379,1389,6144

once/week; cheese 1piece/day; milk type 1 glass/day if consumption of any type of milk; Flora Pro-Active/ Benecol, soft margarine -, olive oil based -, polyunsaturated/sunflower oil based -, other low/reduced fat spread 1 serving/day if in combination with eating at least 2 slices of bread; white, brown, other bread slices 1 slice/day; biscuit, other cereals 1 bowl/day; processed meat 1 piece/day; 0 pieces/day if indicated having never eaten meat poultry/beef/lamb or mutton/pork once/week.

Table S2 Sensitivity Analysis

Transition	Healthy life score		
	0-1 points	2 points	3-4 points
**Exclusion criteria:** Excluding the deaths due to CVD			
Baseline → Diabetes	1.0	0.978(0.948,1.009)	0.965(0.934,0.997) *
Baseline → Death	1.0	0.696(0.666,0.727) *	0.538(0.512,0.566) *
Diabetes → complication	1.0	0.904(0.855,0.955) *	0.870(0.818,0.924) *
Diabetes → Death	1.0	0.724(0.562,0.932) *	0.747(0.567,0.984) *
Complication → death	1.0	0.834(0.637,1.093)	0.790(0.561,1.111)
**Exclusion criteria**: Excluding the follow-up time<2 years			
Baseline → Diabetes	1.0	0.982(0.952,1.012)	0.966(0.935,0.998) *
Baseline → Death	1.0	0.686(0.657,0.715) *	0.543(0.517,0.570) *
Diabetes → complication	1.0	0.907(0.860,0.959) *	0.869(0.818,0.924) *
Diabetes → Death	1.0	0.750(0.591,0.951) *	0.711(0.545,0.928) *
Complication → death	1.0	0.922(0.729,1.166)	0.830(0.614,1.121)
**Exclusion criteria:** Excluding the populations with missing covariates			
Baseline → Diabetes	1.0	0.981(0.951,1.011)	0.966(0.935,0.998) *
Baseline → Death	1.0	0.682(0.655,0.709) *	0.528(0.504,0.553) *
Diabetes → complication	1.0	0.906(0.858,0.957) *	0.869(0.818,0.923) *
Diabetes → Death	1.0	0.752(0.595,0.951) *	0.765(0.591,0.990) *
Complication → death	1.0	0.887(0.706,1.113)	0.849(0.636,1.134)

Table S3 Exposure-response associations between healthy lifestyle and different transitions of diabetes.

Transition	Healthy life score		
	HR	95%CI	P value
**Basic model**			
Baseline → Diabetes	1.00	(0.995, 1.016)	0.323
Baseline → Death	0.765	(0.753, 0.778)	$<2\times10^{-16}$
Diabetes → complication	0.884	(0.868, 0.900)	$<2\times10^{-16}$
Diabetes → Death	0.829	(0.770, 0.893)	7.85×10^{-7}
Complication → death	0.842	(0.777, 0.912)	2.32×10^{-5}
**Model 2**			
Baseline → Diabetes	0.974	(0.962, 0.987)	5.22×10^{-5}
Baseline → Death	0.752	(0.739, 0.765)	$<2\times10^{-16}$
Diabetes → complication	0.913	(0.892, 0.934)	1.16×10^{-14}
Diabetes → Death	0.896	(0.811, 0.990)	0.031
Complication → death	0.925	(0.833, 1.026)	0.139
